# Red Blood Cell Metabolism In Vivo and In Vitro

**DOI:** 10.3390/metabo13070793

**Published:** 2023-06-27

**Authors:** Angelo D’Alessandro, Alkmini T. Anastasiadi, Vassilis L. Tzounakas, Travis Nemkov, Julie A. Reisz, Anastsios G. Kriebardis, James C. Zimring, Steven L. Spitalnik, Michael P. Busch

**Affiliations:** 1 Department of Biochemistry and Molecular Genetics, University of Colorado Anschutz Medical Campus, Aurora, CO 80045, USA; travis.nemkov@cuanschutz.edu (T.N.); julie.haines@cuanschutz.edu (J.A.R.); 2 Laboratory of Reliability and Quality Control in Laboratory Hematology (HemQcR), Department of Biomedical Sciences, School of Health & Caring Sciences, University of West Attica (UniWA), 12243 Egaleo, Greece; aanastasiadi@uniwa.gr (A.T.A.); akrieb@uniwa.gr (A.G.K.); 3 Department of Biochemistry, School of Medicine, University of Patras, 26504 Patras, Greece; vtzounakas@upatras.gr; 4 Department of Pathology, University of Virginia, Charlottesville, VA 22903, USA; jcz2k@virginia.edu; 5 Columbia University Irving Medical Center, New York, NY 10032, USA; ss2479@cumc.columbia.edu; 6 Vitalant Research Institute, San Francisco, CA 94105, USA

**Keywords:** red blood cell, erythrocyte, transfusion medicine, storage lesion, hemolysis, spleen, mitochondria, iron, hematology

## Abstract

Red blood cells (RBC) are the most abundant cell in the human body, with a central role in oxygen transport and its delivery to tissues. However, omics technologies recently revealed the unanticipated complexity of the RBC proteome and metabolome, paving the way for a reinterpretation of the mechanisms by which RBC metabolism regulates systems biology beyond oxygen transport. The new data and analytical tools also informed the dissection of the changes that RBCs undergo during refrigerated storage under blood bank conditions, a logistic necessity that makes >100 million units available for life-saving transfusions every year worldwide. In this narrative review, we summarize the last decade of advances in the field of RBC metabolism in vivo and in the blood bank in vitro, a narrative largely influenced by the authors’ own journeys in this field. We hope that this review will stimulate further research in this interesting and medically important area or, at least, serve as a testament to our fascination with this simple, yet complex, cell.

## 1. Red Blood Cell Metabolism: The Central Role of Oxygen

By recent estimates, the adult human body contains ~30 trillion cells [1,2]. Approximately 90% of these cells are derived from the hematopoietic lineage, of which the overwhelming majority are red blood cells (RBCs) [3]. The 25 trillion circulating RBCs in an adult account for ~83% of all host cells, making RBCs a type of circulating organ critical for human health [4]. The stability of the number of circulating RBCs is ensured by a delicate equilibrium between de novo erythropoiesis and erythrophagocytosis by splenic and hepatic macrophages, which recycle RBC contents, especially iron, proteins and lipids [5].

The evolution of human RBCs has maximized their capacity to transport and deliver oxygen to tissues via progressive loss of nuclei and organelles during the maturation of erythroid precursors to reticulocytes and, ultimately, mature discocytic RBCs [6]. As a result of this process, each mature RBC contains ~250–270 million copies of hemoglobin [7], with hemoglobin accounting for ~98% of the cytosolic proteome and 92% of the total proteome [8]. At full oxygen saturation, RBCs can theoretically carry up to 1 billion molecules of oxygen/cell, a function that is facilitated by the presence of all mature RBCs combined with ~2.6 g of iron (66% of the total body iron) [4]. Fenton and Haber–Weiss chemistry constantly generate the formation of hydrogen peroxide and reactive oxygen species [9,10], making the RBC’s 120 days circulatory lifespan a struggle against the oxidation [11] of proteins (especially redox-sensitive functional residues in hemoglobin such as C93 and H92 of the beta chain [12]), small molecule metabolites [13] and lipids [14]. Every day, 0.2 trillion RBCs are removed from the bloodstream and replaced by de novo erythropoiesis (2 million RBCs are produced per second [15]), accounting for ~40% of the total body mass turnover despite the small mass of each RBC (in the range of 100–300 pg) [3].

The relative simplicity of RBCs, as historically perceived, has attracted multiple efforts to leverage them as a model of simplified human cell metabolism. Indeed, RBCs exclusively rely on glycolysis (Embden–Meyerhof–Parnas pathway) to generate high-energy phosphate compounds, such as adenosine triphosphate (ATP)—whose main source in other cells is oxidative phosphorylation in mitochondria. ATP and its guanosine triphosphate equivalent GTP substantially fuel all key processes in mature RBCs, including: hemoglobin allostery [16,17] to metabolism [18], from proton pumps [19] to membrane integrity by phosphorylating structural proteins [20], from protein stabilization via fueling of transglutaminase 2 [21] to cellular mechanics [22], from cytoskeletal actin polymerization [23] to vesiculation [24], from membrane lipid symmetry by fueling phosphatidylserine flippases [25] to proteasomal activity to remove damaged proteins [26,27,28]. Ultimately, the energy-depleted erythrocyte is rapidly lost from the bloodstream [29] via intra- or, more commonly, extravascular hemolysis through splenic sequestration and erythrophagocytosis.

Two critical functional RBC pathways branch from glycolysis: the Rapoport–Luebering shunt, which generates 2,3-diphosphoglycerate (DPG), and the pentose phosphate pathway (hexose monophosphate shunt), which generates ribose phosphate and, importantly, reduced nicotinamide adenine dinucleotide phosphate (NADPH). DPG is a critical allosteric modulator of hemoglobin, promoting oxygen release from hemoglobin to counteract hypoxia (e.g., at high-altitude [30] or from hemorrhage [31]). NADPH fuels multiple antioxidant processes in RBCs [32]: (i) it is essential for the reduction in oxidized glutathione by glutathione reductases; (ii) it directly or indirectly fuels glutathione peroxidase 4 [33], catalase, peroxiredoxins [34], glutaredoxins, the thioredoxin reductase system, biliverdin reductase B [35], the ascorbate-tocopherol axis [36] and other diaphorases such as NADPH-dependent quinone oxidoreductases (NQO1) [37]. NAD(P)H-dependent methemoglobin reductases are also essential for converting (auto-oxidized) ferric hemoglobin iron back to its ferrous state. The C93 residue of hemoglobin beta also participates in antioxidant systems by buffering free glutathione [38], participating in recycling oxidized peroxiredoxin 2 [39], and contributing to nitrite reduction [40] (resulting in the pathological generation of methemoglobin in the setting of nitrite poisoning [41]). Owing to its role in redox chemistry, it has also been proposed that hemoglobin may serve as a murzyme (i.e., a redox enzyme working along the principles of the Murburn concept), thereby contributing to ATP synthesis [42].

The rate-limiting enzyme of the pentose phosphate pathway is glucose 6-phosphate dehydrogenase (G6PD). G6PD is encoded by a gene on chromosome X. Mutations of this gene are found in >500 million people around the world, with >200 G6PD mutations known in humans [43]. Individuals carrying such mutations typically present with a significant loss of enzymatic activity, ranging from <1% in the most severe forms (e.g., the Mediterranean variant-S188F) [44,45] to <10% in the common African variant (V68M); the latter is extremely common in some metropolitan areas such as New York, especially in African American communities (~13% prevalence) [46]. In China, the six most common mutations account for ~90% of G6PD-deficient alleles, with an overall national prevalence of ~2.10% [47]. Human and mouse RBCs carrying these mutations are extremely susceptible to hemolysis following oxidant insults [48,49]. As in the case of hemoglobinopathies, such as sickle cell trait [50] and beta-thalassemia [51], positive selection for these mutations in human populations is thought to be associated with the selective pressure by malaria infections in the Mediterranean and South East Asia areas, with considerable overlap between the incidence of G6PD deficiency and malaria-endemic regions [52]. Protection against mild malaria infection is also observed in heterozygous G6PD deficient females [52]. G6PD deficiency, sickle cell trait (or disease) and beta-thalassemia (minor or major) are all associated with RBC metabolic reprogramming consistent with increased susceptibility to oxidant stress-induced hemolysis (e.g., by quinone antimalarials and sulfa drugs in the setting of G6PD deficiency).

The regulation of glycolysis by oxidant stress to functional thiols in rate-limiting enzymes, including glyceraldehyde 3-phosphate dehydrogenase (GAPDH) at cysteine residues 152 and 156 [53] and pyruvate kinase [54,55], provides a strategy to constrain metabolic fluxes through late glycolysis when oxidant stress is high while redirecting glucose oxidation to the pentose phosphate pathway to produce reducing equivalents that counteract this stress. Similarly, the most abundant RBC membrane protein, band 3 (or, equivalently, anion exchanger 1—AE1) has a very acidic N-terminus cytosolic domain that can serve as an inhibitory docking site for glycolytic enzymes at high oxygen saturation; in contrast, at low oxygen saturation deoxyhemoglobin outcompetes the glycolytic enzymes, displacing them from the membrane and boosting glycolysis to stimulate ATP and DPG production in the face of hypoxia [56,57]. Prolonged, unmitigated oxidant stress, such as during refrigerated RBC storage under blood bank conditions, promotes proteolytic or reactive oxygen species (ROS)-triggered proteolysis of the N-terminus of band 3, ultimately causing the loss of this RBC oxygen-dependent metabolic modulation pathway [58,59,60]. In addition, genetic mutations of the N-terminus region of band 3 are associated with severe hemolysis and necessitate lifelong transfusion [59].

## 2. RBC Metabolism beyond Glycolysis

**Figure 1:**
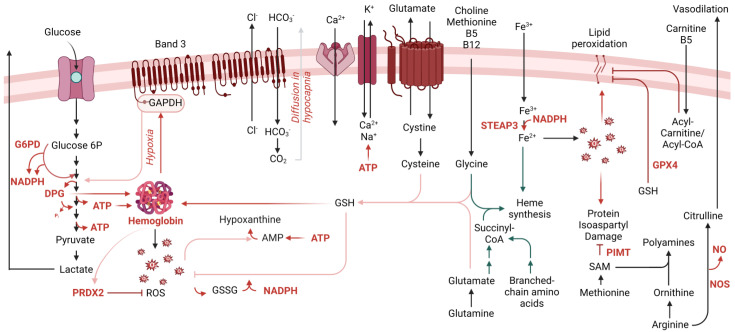
Overview of the main metabolic pathways relevant to RBC physiology during aging in vivo and in vitro.

Over the last 50 years, almost all computational efforts to simulate the RBC metabolome ex vivo were limited to the pathways described above [61,62,63]. The recent implementation of omics technologies to study RBCs has revealed an unanticipated complexity of the erythrocyte proteome, now counting ~2500–3000 unique proteins; indeed, functional metabolic tracing experiments suggest that even this list may be incomplete [8,59,64,65,66,67,68,69]. Leveraging these recent datasets, systems biology experts are redrawing connectivity maps of the human RBC metabolome (Figure 1), of relevance for basic science and translational applications [70,71]. With >77 active transporters, circulating RBCs can take up and release many metabolites from peripheral tissues, making RBCs a unique window into system health [72]. From creatinine and carnitine (as markers of renal function [73]) to conjugated bile acids (from the gut microbiome [74]), from transamination products (e.g., alanine, glutamate, aspartate) to neurotransmitters (e.g., serotonin, dopamine, acetylcholine), RBCs can directly and indirectly participate in systems metabolism throughout the body.

During erythropoiesis, amino acid metabolism (e.g., glycine, glutamine, branched-chain amino acids) is essential to heme synthesis as both direct and indirect substrates (e.g., succinyl-CoA from glutaminolysis and branched-chain amino acid catabolism). This process concomitantly releases ammonium, making erythropoiesis and de novo glutamine synthesis two of the leading pathways contributing to ammonium homeostasis in humans [75]. Genetic defects of rate-limiting genes in the branched-chain amino acid catabolism (e.g., in propionic [76] or methylmalonic acidemias) are associated with defects in erythropoiesis [77]. Along with cysteine, both glycine and glutamine-derived glutamate contribute to the synthesis of the glutathione tripeptide. The exchange of cystine—a cysteine disulfide—to glutamate is a critical regulator of a process previously referred to as eryptosis [78] and, more recently, recognized as ferroptosis [79] owing to its expanded relevance in other cells besides RBCs.

When considering erythropoiesis, one-carbon metabolism is one of the first pathways that is referenced. Folates and methyl group donors such as methionine, choline and betaine—along with B6, B12 and B5 cofactors [80,81]—are essential for de novo purine nucleotide synthesis to support proliferation, as well as for generating glycine to contribute to heme synthesis described above. However, methyl-group donors also participate in RBC redox homeostasis and its dysregulation, such as in the context of folate dietary deficiency or excess [81]. This is also relevant in diseases associated with alterations of this pathway such as homocystinuria [82] or Down syndrome [83], owing to gene dosage of cystathionine beta synthase resulting in a “folate trap”-like phenotype [84]. These conditions present with macrocytic or megaloblastic anemia [82]. In addition, in keeping with the central role of oxidant stress in the economy of RBC metabolism, methionine uptake and consumption in RBCs fuel a pathway of isoaspartyl protein damage repair. As a hallmark of RBC aging in vivo [85], deamidation of asparagine residues in key structural membrane proteins and glycolytic enzymes [86,87,88,89,90,91,92] alters the protein backbone, with important structural/functional implications. Since RBCs cannot replace damaged components by de novo protein synthesis, repairing this damage is essential to their survival. The methylation of deamidated residues favors the formation of a succinimide intermediate, ultimately promoting the rescue of the protein backbone structure in 15–30% of the cases, a reaction catalyzed by the enzyme “protein isoaspartyl o-methyltransferase” (PIMT). Increased uptake of methyl donors such as methionine is observed in RBCs in response to oxidative challenges (e.g., incubation with hydrogen peroxide [93], refrigerated storage under blood bank conditions [92]). Thus, one can speculate that RBC uptake and consumption of methyl-group donors (e.g., methionine, choline) could compete with other tissues where these substrates are used to fuel epigenetic regulatory mechanisms, such as methylation of proteins (e.g., histones), RNA (N6-methyladenosine) and DNA (CpG islands), making RBCs an indirect player in systemic long-term responses to oxidant stress events.

In response to oxidant stress, ATP synthesis is reduced because of the redox sensitivity of glycolytic enzymes and the band 3-dependent mechanism, as described above. In this case, ATP breaks down into lower energy phosphate compounds, ADP and AMP, the latter being prone to deamination to IMP by RBC-specific AMP deaminase 3 [94]. Phosphoribolysis of IMP releases hypoxanthine, a substrate for xanthine oxidase to generate xanthine and urate, with concomitant production of hydrogen peroxide [94]. The presence of an active xanthine oxidase in mature RBCs was reported and challenged [95]. Exogenous urate is a potent antioxidant in mature RBCs [96]. Similarly, the breakdown of AMP into adenosine, with adenosine oxidation by adenosine deaminase was reported as contributing to hypoxanthine accumulation [97]. The recycling of hypoxanthine by X-linked hypoxanthine guanosine phosphoribosyltransferase (HPRT) preserves IMP/GMP homeostasis in RBCs. Lesch Nyhan syndrome patients exhibit genetic mutations of this enzyme and present with a range of clinical manifestations, including macrocytic anemia [98]. Purine homeostasis is relevant to RBC homeostasis for additional reasons beyond those described above. For example, ATP release by RBCs has regulatory effects on endothelial cells and on the RBCs themselves, as a type of autocrine signaling. The breakdown of extracellular ATP to ADP, AMP and adenosine by ectonucleotidases (e.g., CD38) generates agonists of P2Y receptors on endothelial cells, but adenosine can also be imported by equilibrative nucleotide transporters (ENT1) or stimulate adenosine receptors (e.g., ADORA2b). Both of these pathways contribute to RBC responses to hypoxia, the former, for example, by limiting circulating adenosine, a process that is counteracted by irreversible ENT1 degradation to facilitate acclimatization to high altitude hypoxia upon reascent [99]. In addition, ADORA2b transduces an intracellular signaling pathway that activates downstream protein kinase A and AMP-dependent kinase (AMPK), both contributing to metabolic reprogramming; for example, via phosphorylation-mediated activation of bisphosphoglycerate kinase [97,100].

Recent applications of unsupervised metabolomics and lipidomics approaches identified a sphingolipid sphingosine 1-phosphate (S1P) as a relevant metabolite regulating RBC responses to hypoxia [101]. For example, in response to high altitude-induced hypoxia [101] S1P can bind to deoxyhemoglobin following its stabilization by DPG, further promoting oxygen off-loading [102]. By further stabilizing deoxyhemoglobin, S1P also contributes to energy metabolism by promoting the release of glycolytic enzymes from band 3 into the cytosol, with subsequent activation of glycolysis at the expense of the pentose phosphate pathway. Although this adaptation is beneficial in hypoxia, it is deleterious when RBCs are challenged by oxidant stress, such as during refrigerated storage in the blood bank [103] or in sickle cell disease, where deoxy-sickle hemoglobin stabilization further promotes its crystallization [102].

Using a combination of state-of-the-art multi-omics technologies, we recently identified the presence and activity of oxidant stress-sensitive fatty acid desaturases (especially FADS2) in mature RBCs [104]. By introducing double bonds in an NADH-dependent fashion, FADS contribute to NADH homeostasis by recycling reducing equivalents back to their oxidized state, which is essential for the glycolytic step catalyzed by GAPDH. Of note, alterations of this pathway are critical for regulating abnormal hematopoiesis in aging mice and humans [105]. Dietary interventions that alter fatty acyl membrane composition predispose, or protect, lipids from peroxidation [106], a hallmark of ferroptosis that regulates RBC extravascular hemolysis; the latter is—at least in part modulated by the activity of the ferrireductase STEAP3 [107]. Pathways exist in mature RBCs to cope with lipid peroxidation; for example, via glutathionylation by *GPX4*—which is present and active in mature erythrocytes [33]. Genetic polymorphisms in the *GPX4* coding region are common in humans and are associated with an increased propensity to hemolysis following oxidant stress [49]. Alternatively, phospholipase A2 (or PLA2-like enzymes such as peroxiredoxin 6 [108]) can release the oxidized fatty acid moiety from membrane phospholipids, thereby generating lysophospholipids. The acyl-coA/acyl-carnitine system can then fuel the transfer of new fatty acids to the lysophospholipid, restoring phospholipid composition. Carnitine availability limits the rate of this pathway, known as the Lands cycle, which is activated in response to oxidant stress (e.g., in response to exercise [109] or in patients with hemoglobinopathies, such as sickle cell trait [110] and sickle cell disease [111,112]).

Arginine metabolism in mature RBCs was found to be more complex than anticipated, despite the absence of mitochondria, where a subset of critical reactions in the urea cycle are known to occur. For example, RBCs contain high levels of arginase 1 [113], which converts arginine to ornithine, a precursor of polyamines via ornithine decarboxylase. Although this pathway is critical in erythropoiesis, owing to the role of polyamines in regulating the intracellular pH of hematopoietic precursors [114], in adult RBCs this pathway has been associated with cellular responses to iron-deficient anemia and abiotic stresses, such as exposure to radiation [115]. Arginine metabolism cross-talks with heme synthesis, glutathione homeostasis and one-carbon metabolism, in that polyamine synthesis and creatine synthesis are both affected in human RBCs by factors such as sex and age [48], and compete for rate-limiting substrates for the generation of each product. During the last decade, RBCs were found to harbor a functional nitric oxide synthase [116], which converts arginine to citrulline and concomitantly generates nitric oxide, a potent vasodilator with a crucial role in the regulation of endothelial cells and related vascular function [117,118].

As already mentioned above, carboxylic acid intermediates of the Krebs cycle, such as succinyl-CoA participate in erythropoiesis by fueling heme synthesis. In addition, small molecule dicarboxylates in this pathway (e.g., succinate, fumarate, malate) sustain erythropoiesis by the mechanism of stabilization of the Hypoxia Inducible Factor 1alpha upon exposure to hypoxia, which otherwise is degraded following hydroxylation by alpha-ketoglutarate-dependent prolyl hydroxylases [119]. The conversion of alpha-ketoglutarate to 2-hydroxyglutarate can occur under hypoxic conditions by non-canonical lactate dehydrogenase activity [120], and testosterone-induced stimulation of erythropoiesis [121]. The absence of mitochondria in healthy mature RBCs originally led the field to believe that minimal carboxylic acid metabolism would occur in this cell system. However, proteomics recently identified functional cytosolic isoforms of acetyl-CoA ligase, isocitrate dehydrogenase 1, malate dehydrogenase 1 and malic enzyme 1; thus, catabolism of pyruvate and citrate can occur in mature RBCs [122,123] via a series of reactions that can fuel the alternative generation of reducing equivalents (e.g., NADPH and NADH), especially in hypoxia [124]. The activation of these pathways may be especially beneficial as a partial compensatory mechanism in the face of genetic aberrations leading to a dysfunctional pentose phosphate pathway, such as G6PD deficiency [46,125].

Although healthy mature RBCs are indeed devoid of mitochondria, recent evidence unequivocally documented the presence of up to 6–7 mitochondria per cell in mature RBCs from sickle cell patients [126,127,128,129]. Similar observations have been reported in the context of other pathophysiological conditions, such as systemic lupus erythematosus [130] and Rett syndrome [131,132]. Whether and to what extent these organelles still function is incompletely understood, though their retention might result from a defect in mitophagy or the ubiquitin-proteasome system [126,127,132] Nonetheless, the presence of metabolically active mitochondria in mature RBCs could induce oxygen consumption and consequently oxidant stress through ROS generation [128]. The induction of reticulocytosis or heterogeneous retention of mitochondria is associated with alloimmunization in a murine model of transfusion [133], which could be relevant to blood donors with heterogeneous mitochondrial DNA content in circulating erythroid cells and recipients with inflammatory conditions such as systemic lupus erythematosus [131]. The relevance of this phenomenon in RBC aging in vivo—to the extent this may result from/contribute to processes of age-related aberrant erythropoiesis and age-related comorbidities remains to be determined. Nonetheless, one may speculate that intra- or extra-vascular hemolysis of mitochondria-containing RBCs may release prokaryotic-like RNA and DNA into the circulation or in phagocytic macrophages, thereby triggering cGAS-STING-Interferon responses [130,134], ultimately driving interferon-mediated inflammatory complications in sickle cell patients, lupus patients and in other conditions in which interferonopathies and hematological anomalies are observed (e.g., Down syndrome) [135].

## 3. RBC Metabolism and Blood Storage for Clinical Transfusion Purposes

Understanding RBC metabolism holds critical translational implications in modern medicine. Transfusion of packed RBCs is a life-saving intervention for 4–5 million Americans every year. With over 110 million units of packed RBCs collected and transfused annually worldwide, RBC transfusion is the most common hospital iatrogenic intervention after vaccination [136]. Storage in the blood bank for up to 42 days in most countries is a logistic necessity to make RBC units available for transfusion to acutely or chronically ill recipients, such as those with trauma [137] or beta-thalassemia/sickle cell disease [138], respectively. Unfortunately, during refrigerated storage under blood bank conditions, RBCs undergo a series of biochemical, metabolic and morphological changes, collectively referred to as the “storage lesion” [139]. Application of omics technologies to the investigation of the metabolic storage lesion has documented a plethora of changes [140], with a temporal sequence of events [141] first started by slower kinetics of metabolic enzymes at 4 °C [142]. As temperature-sensitive ion pumps fail under refrigerated storage conditions, increased ATP demands to counteract these effects through the active transport of potassium and calcium ions against gradients [143] results in additional metabolic strains for RBC glycolysis. While glucose consumption and the generation of lactate are still observed in stored erythrocytes, the rate at which these fluxes occur is insufficient to meet the demand for ATP and DPG of refrigerator-stored RBCs [144]. Slow glycolytic rates are aggravated by multiple mechanisms beyond storage temperature. First, in the closed system of a blood bag, accumulation of lactic acid for up to 42 days is accompanied by the progressive acidification of the intracellular and extracellular pH, ultimately leading to slower kinetics for pH-sensitive enzymes, such as phosphofructokinase, bisphosphoglycerate mutase and G6PD, rate-limiting enzymes of glycolysis, the Rapoport–Luebering shunt and the pentose phosphate pathway, respectively [145]. Strategies have been envisaged to counteract this phenomenon, such as the development of alkaline additives with low/no chloride and high bicarbonate content [146,147]. As ATP and DPG are consumed, the latter >95% depleted by storage weeks 2–3 [148], and RBC oxygen saturation increases up to 95% by storage day 21, with concomitant accumulation of ROS [141]. ROS attack on functional residues of hemoglobin and glycolytic enzymes such as GAPDH further negatively affects glycolytic fluxes, resulting in the transient activation of the PPP to generate NADPH and counteract storage-induced oxidant stress [53]. These antioxidant systems are insufficient to cope with oxidant stress, ultimately resulting in the irreversible oxidation of functional enzymes and structural proteins, such as band 3, ankyrin and spectrin [141,149], with consequent alteration of the membrane band 3 interactome [59]. Mechanisms of isoaspartyl protein damage methylation—such as the ones described above as a function of PIMT activity in aging RBCs in vivo—are activated to cope with oxidant stress to structural proteins and glycolytic enzymes in stored RBCs [92]. However, calcium-activated caspase and oxidative stress both contribute to the fragmentation of the N-terminus of band 3 [150], ultimately depriving RBCs of the capacity to inhibit GAPDH (and other glycolytic enzymes) by mechanism of inhibitory binding to this region [59,60]. Interestingly, a similar failure of this mechanism is observed upon exposure to chronic oxidant stress in vivo in sickle cell disease [151]. Since fragmentation to band 3 is irreversible, and no new band 3 protein can be synthesized, stored transfused RBCs cannot respond to oxidant stress in vivo by activating the pentose phosphate pathway at the same rate that fresh RBCs would do—as observed in storage-biotinylation-recovery studies in humans [152]. Interestingly, transient activation of the pentose phosphate pathway at the second week of storage is a measurable transient metabolic state in the unsupervised elaboration of omics data of stored RBCs [70]. The activation of glycolysis at the expense of the pentose phosphate pathway by exogenous supplementation (or inter-donor heterogeneity in the levels at donation) of S1P is associated with an increased susceptibility of stored RBCs to extravascular hemolysis [103]. Genetic ablation of the S1P synthesizing enzyme Sphk1 in mice improves storability and post-transfusion recovery (PTR) [103]. Polymorphisms in the S1P transporter Mtfsd2b are relatively common in the blood donor population and are associated with increased susceptibility to osmotic fragility [49].

In RBCs obtained from donors with non-hemolytic G6PD deficiency, whose RBCs have naturally aberrant flux through the pentose phosphate pathway at baseline, storage results in higher basal levels of glycolysis and ATP, despite increased oxidant stress; this unusual combination yields RBCs that have better-preserved morphology by the end of the storage period [44,45] yet suffer from an exacerbated redox storage lesion, ultimately resulting in increased susceptibility to storage, osmotic and oxidant stress-induced hemolysis [48,49], as well as poorer PTR, i.e., the percentage of stored RBCs that still circulates at 24 h upon transfusion. As the ATP synthesis rate does not meet demand, lower energy phosphate compounds such as AMP accumulate and are deaminated by AMPD3 into IMP and hypoxanthine, a phenomenon exponentially activated after storage week 3 [94]. The timeline is not casual, in that by storage day 21 we observe a peak in consumption of DPG, with concomitant significant accumulation of intracellular calcium—both factors promoting AMPD3 activity [94]. The accumulation of hypoxanthine, a biomarker of the RBC metabolic storage lesion and a predictor of PTR in stored human and murine RBCs, is observed not just in human RBCs, but also in other primates (macaques [153], baboons [154]) and mammals (e.g., mouse [155], rats [156], guinea pigs [157], cows, dogs, donkey and horses [158]), though at different rates as a function of species genotypes, suggesting a genetic regulation of this pathway—which is relevant for veterinary transfusion considerations [159]. Since HPRT (see above) is an X-linked gene, sex dimorphisms in this pathway are observed in the stored RBCs [48]. One additional confounder related to sex pertains to the relative age of circulating RBCs at the time of donation, generally younger in pre-menopausal females [160], further confirming a sex dimorphism in RBC storability [161]. Indeed, small-scale studies have demonstrated RBC storability differences in the hemolytic propensity and membrane binding of stress protein markers between pre- and post-menopausal women [162,163]. Similarly, and still part of the purine oxidation pathway, inter-donor heterogeneity in uric acid levels is observed and contributes to the overall variability in antioxidant capacity across blood units [96].

Ultimately, unmitigated oxidant stress contributes to increased fatty acid desaturation, oxidation and migration to the membrane of oxidized proteins (e.g., peroxiredoxin 2 [34]), vesiculation of oxidized proteins [12] and lipids [164], a process that promotes the loss of the RBC discocytic phenotype and the acquisition of an echinocytic, spheroechinocytic and spherocytic morphology [141,143,165,166]. Losing cell volume increases the surface-to-volume ratio, making the small microcytic erythrocyte (<43 µm^2^) less deformable and more susceptible to sequestration in the splenic slits, ultimately priming erythrophagocytosis. It should be noted that the extracellular vesicles that accumulate in stored RBC supernatant could act as biological response modifiers, potentially affecting post-transfusion responses, especially in light of recent studies that demonstrate the presence of several RNA transcripts in RBC-derived vesicles [167].

The implementation of high-throughput metabolomics technologies is now informing the data-driven development of novel storage additives [168]. In the meantime, the summary above generally holds true for RBCs stored in almost all currently licensed storage additives, from saline adenine glucose mannitol (SAGM), to additive solution (AS) 1, 3, 5, and phosphate adenine glucose gluconate saline mannitol (PAGGSM), with slightly different kinetics [145,169,170,171,172]. The data are so consistent that biomarkers of the metabolic storage lesion have been calculated [70] and used to predict stored RBC metabolic states throughout the time course even from just a single data point [173]. Heterogeneity in blood storage solutions is indeed a critical parameter modulating the storage lesion, one that explains almost the same percentage of the total metabolic variance across stored units as storage duration itself [174].

Nonetheless, the extent to which these storage-induced changes correlate with functional and clinically relevant outcomes is only partially understood. For example, it is now evident that DPG and ATP depletion perfectly predict alterations in RBC oxygen kinetics, with faster oxygen binding and slower oxygen off-loading kinetics [18]. Of note, RBC storage under hypoxic and hypocapnic (reduced carbon dioxide) conditions promotes intracellular alkalinization by boosting the exchange of chloride for bicarbonate (strong vs. weak acid) via carbonic anhydrase/band 3 activity—which is dependent on residues 559–630 and 681 as per Uniprot entry P02730—phenocopying the benefits of alkaline additives [175]. In so doing, hypoxic storage boosts RBC DPG levels [176], preserving oxygen kinetics of the stored RBC [177]. At the same time, hypoxic storage deprives the system of oxygen, a substrate for the chemistry driving the formation of ROS, ultimately mitigating PTR of murine [178] and human RBCs [179]. It is tempting to hypothesize that improved ATP preservation under hypoxic conditions [177,179] could additionally benefit stored RBCs through enhanced proteostatic regulation, including efficient chaperoning and proteasome activity, a feature that is crucial for a cell without the ability to produce new protein molecules.

## 4. All Blood Units Are Created Equal, but Some Blood Units Are More Equal than Others

**Figure 2:**
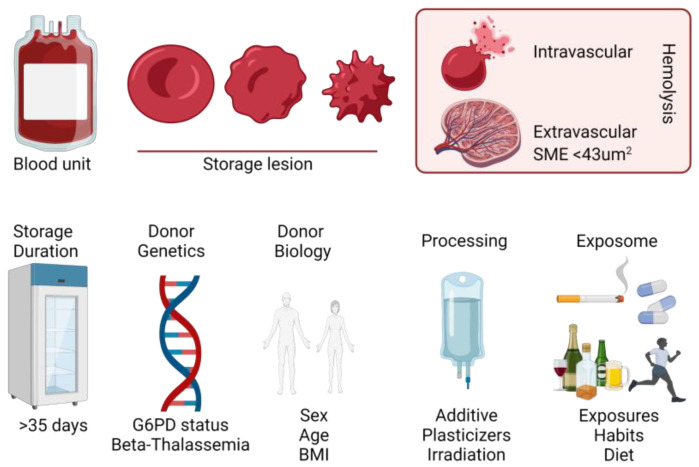
Summary of the main factors impacting RBC storage metabolism and post-transfusion performances.

A meta-analysis of multiple PTR studies in healthy volunteers has clearly shown that the quality of donated blood is heterogeneous across donors [180] (Figure 2). In recent years, a series of studies have shed light on inter-donor heterogeneity that manifests itself in variable hemolytic propensity and post-transfusion hemoglobin increment as a function of donor biology, including donor sex, age, ethnicity and body mass index/obesity as well as genetic mutations in enzymes or Hb [48,51,181,182,183]. Further studies in monozygotic (identical) and dizygotic (non-genetically identical) twins have shown that hemolysis and metabolite levels, especially of antioxidants such as glutathione, are heritable in the blood donor population [184,185,186]. Leveraging this concept, studies in murine models of blood storage and PTR have clearly shown cross-strain heterogeneity in extravascular hemolysis [155,187].

Elegant breeding strategies were executed to cross good and poor storing mouse strains, ultimately leading to the identification of the ferrireductase STEAP3 as a critical mediator of lipid peroxidation and extravascular hemolysis of stored, transfused murine RBCs [107]. The rationale driving the aforementioned studies is now being expanded to large-scale studies in humans, with investigations such as the Recipient Epidemiology and Donor Evaluation Study (REDS) [188,189]. As part of this study, ~14,000 volunteers were enrolled to donate blood at four different blood centers across the United States. Units were stored for up to 42 days and, at the end of the storage period, they were tested for spontaneous hemolysis or hemolysis induced by oxidative or osmotic insults [190]. Donors with extreme hemolytic propensity (5th and 95th percentile) were invited to donate a second unit of blood, which was tested again for the same parameters, showing significant reproducibility of intra-donor hemolytic propensity across multiple donations [190]. These donors were genotyped for 879,000 Single Nucleotide Polymorphisms (SNPs) [191], which identified genetic underpinnings of hemolytic propensity, including G6PD and GPX4 status, among others [49]. The linkage of genetic polymorphisms, hemolytic propensity, and hemoglobin increments in recipients of units from these donors via a vein-to-vein linkage database [181,192] and metabolite levels (metabolite quantitative trait loci—mQTL) [193] is currently underway, and early results are already significantly expanding our understanding of the impact of donor genetics on RBC metabolism during aging in vitro in the blood bank on in vivo RBC recovery and function following transfusion.

Beyond donor genetics, metabolites were identified that do not change with storage, but are rather associated with donor exposures—the so-called exposome, factors that are now being associated with hemolytic propensity [194]. From these studies, it is emerging that donor habits, such as smoking or other nicotine exposures, consumption of alcohol, coffee and caffeinated, taurine-rich beverages, all impact RBC energy and redox metabolism in a way that affects storage biology and, potentially, post-transfusion performance, especially when combined with invasive processing such as RBC unit irradiation [195,196,197,198]. While phthalate plasticizers were historically added to polyvinylchloride bags to decrease the rigidity of the blood bag, these compounds leach from the unit and intercalate into the RBC membranes, resulting in an erythrocyte with altered deformability, decreased hemolytic propensity [199,200], and altered increased risk for toxicity (e.g., infertility, cardiodepression), especially in certain categories of recipients such as pediatric patients [201]. Controversial reports on the detection of metabolites of professional exposures such as Perfluoroalkyl and Polyfluoroalkyl Substances (PFAS) in high-fidelity recurring donors such as firefighters have been described [202], though the potential toxicity profiles of these compounds in transfusion recipients remain to be assessed. Similarly, a long list of over-the-counter or prescription drugs that are not grounds for blood donor deferral have been detected, at least in traces, in blood units, including acetaminophen/paracetamol, ibuprofen, statins, xartans, proton pump inhibitors, antidepressants, just to mention few [194]. Inter-donor heterogeneity in diets results in differential lipid composition of RBC membranes, which ultimately affects membrane fluidity and fragility [203,204,205]. Other antioxidant molecules of dietary origin such as carnitine, vitamin C, vitamin E, resveratrol, quercetin and ergothioneine have all been detected at variable levels in donated blood and could theoretically affect RBC storability [194,206,207,208,209,210,211,212]. While exercise is known to significantly impact RBC metabolism and deformability [109], little is known as to whether RBCs from athletes store and recover better than RBCs from sedentary donors. Similar considerations can be made for blood donors living at high altitudes vs. sea level [30]. RBCs from subjects previously infected with corona- or flavi-viruses such as SARS-CoV-2, present metabolic alterations consistent with anomalous activation of the pentose phosphate pathway and band 3 oxidation/fragmentation [213,214]. In the case of Zika virus-infected donors, clearance of viremia and seroconversion were still accompanied by months-long (up to >100 days) alterations of RBC metabolism, suggesting potential long-lasting effects of infection on circulating RBC biology, perhaps relevant to the blood donors and recipient populations, e.g., in the context of sepsis [215]. Altogether, all the factors listed above contribute to the metabolic heterogeneity of the stored RBC beyond the chronological age of the unit (i.e., the days elapsed since donation), suggesting a more relevant role for the metabolic age of the unit as a critical predictor of transfusion outcomes in the future [216].

Although all these changes are well documented, it is unclear whether and to what extent they are reversed in vivo following transfusion, and whether they ultimately affect RBC performance in vivo. Autologous blood transfusion studies in healthy volunteers have shown that stored RBCs significantly impact healthy recipient plasma metabolism [113], an observation that may inform strategies for autologous blood doping detection in sports [217]. Studies on autologous volunteers and recovery of biotinylated RBCs suggest that part of the storage lesion is reversible in vivo, such as the rescue of ATP and DPG levels, a process that may require up to 24–72 h [152,218] and thus be sufficiently slow that stored RBC transfusions may not correct oxygen kinetics within the golden hour of hypoxic patients undergoing massive bleeding, to mention a key category of recipients [137]. Studies on the metabolic impact of transfusion in massively transfused trauma patients are currently underway [137], while clear evidence of an association with cardiorenal dysfunction and systemic hypoxia has been reported in sickle cell patients [111,138]. Interestingly, studies of ex vivo preservation of murine RBCs have shown that erythrocytes from mice with good or poor storage quality cross-regulate, suggestive of as yet under-investigated mechanisms of metabolic cross-regulation, ultimately impacting post-transfusion performance [219]. It remains to be determined whether these “good apple, bad apple” mechanisms occur in vivo, for example in sickle cell patients undergoing exchange therapy, or even when the donors’ RBCs are exposed to the recipients’ erythrocytes.

As our understanding of RBC metabolism in vivo and in vitro refines, novel pathways are discovered and mechanisms elucidated, the simple cell of the early days of biochemistry keeps surprising us with novel pathways and novel roles in systems metabolism beyond oxygen transport. Multi-omics studies on RBC propensity to hemolysis upon storage and transfusion are finding new mechanisms even for well-investigated proteins such as band 3; for example, while the extracellular domain of this protein has long been recognized as the Diego blood group in transfusion medicine [220], it has only recently emerged that genetic polymorphisms in the region coding for this protein impact RBC hemolytic propensity and post-transfusion performances [49]. It is easy to anticipate a near future where RBCs will no longer be considered an inert background participant in human biology, but rather a targetable vulnerability of human health, longevity and disease, beyond the current focus in transfusion medicine.
